# Nationwide trends in radiotherapy use among older patients with early-stage non-small cell lung cancer in Japan, 2013–2022

**DOI:** 10.1038/s41598-026-44945-z

**Published:** 2026-03-31

**Authors:** Toshiki Ikawa, Kayo Nakata, Kenji Kishimoto, Toshitaka Morishima, Naoyuki Kanayama, Masahiro Morimoto, Koji Konishi, Isao Miyashiro

**Affiliations:** 1 Cancer Control Center, Osaka International Cancer Institute, 3-1-69 Otemae, Chuo-ku, Osaka, 541-8567 Japan; 2 Department of Radiation Oncology, Osaka International Cancer Institute, Osaka, Japan

**Keywords:** Aged, Lung neoplasms, Octogenarian, Radiosurgery, Radiotherapy, Surgery, Cancer, Medical research, Oncology

## Abstract

This study examined trends in radiotherapy use among older patients with early-stage non-small cell lung cancer (NSCLC) in Japan. Using hospital-based cancer registry data (2013–2022), we analyzed 295,662 patients aged ≥ 65 years with epithelial lung tumors (clinical Tis–T2N0M0), excluding those with poorly differentiated endocrine carcinoma or missing treatment data. Overall, 80% underwent surgery, 11% received radiotherapy, and 9% received other management. Radiotherapy use increased from 10.5% in 2013 to 12.8% in 2022 (age- and sex-standardized increase of + 1.2% points). Logistic regression showed increased odds of radiotherapy use in the later period across subgroups according to age, sex, clinical T category, and facility volume, except among patients aged 75–84 years and those treated at high-volume facilities. From 2013 to 2022, radiotherapy use changed by + 1.7, + 0.2, and + 4.5% points among patients aged 65–74, 75–84, and ≥ 85 years, reaching 6.2%, 14.8%, and 35.5%, respectively. Surgery use changed by − 2.1, + 1.3, and − 3.2% points, with values of 89.6%, 76.3%, and 32.8%, respectively. Radiotherapy use increased only modestly, with variation across age groups. Over 30% of patients aged ≥ 85 years underwent surgery, indicating the need to clarify the roles of surgery and radiotherapy in this population.

**Supplementary Information:**

The online version contains supplementary material available at 10.1038/s41598-026-44945-z.

## Introduction

Lung cancer is one of the most common malignancies worldwide and remains a major public health challenge^1^. This pattern is similar in Japan, where, in 2021, lung cancer was the second most frequently diagnosed cancer after colorectal cancer and remained the leading cause of cancer-related death^2,3^. Although the age-adjusted incidence in both sexes has plateaued or declined since around 2010, the absolute number of newly diagnosed patients has remained high, highlighting the persistent burden of the disease in Japan^2,4^.

This sustained burden is partly driven by population aging. Japan has one of the longest life expectancies in the world, with adults aged ≥ 65 years comprising 29% of the population in 2022^5,6^. This demographic structure has contributed to the persistently high number of lung cancer diagnoses and a shift in the age distribution of patients. Between 2016 and 2021, the annual number of lung cancer diagnoses increased among patients aged ≥ 70 years but declined among those aged < 70 years^2^. In addition, the proportion of early-stage (localized) lung cancer has risen, likely reflecting wider adoption of screening and more frequent incidental detection during imaging for other conditions^7,8^. Localized disease was diagnosed in 27% of male and 39% of female patients between 2010 and 2015^7^. These shifts toward an older patient population and more early-stage diagnoses have added complexity to treatment decision-making. In older patients with early-stage disease, despite the high likelihood of cure, age-associated health limitations may require careful balancing of benefit and risk in treatment planning.

Surgical resection is the standard treatment for early-stage non-small cell lung cancer (NSCLC)^9–11^, the predominant histological subtype of lung cancer^7^. However, older patients often have limited physiologic reserve and comorbidities, making surgery unfeasible in some cases. For medically inoperable patients, stereotactic body radiotherapy (SBRT) serves as a curative-intent alternative^9–11^. SBRT delivers high radiation doses in a limited number of fractions, and clinical trials have demonstrated high local control with relatively low toxicity^12,13^. The Japan Clinical Oncology Group (JCOG) 0403 trial also showed favorable outcomes of SBRT for early-stage NSCLC^13^. With the accumulation of clinical evidence and updated guidelines, SBRT use has increased in several countries^14,15^.

In medically operable patients, SBRT has shown promising outcomes^12,16^. In the revised STARS trial, patients with operable early-stage NSCLC treated with SBRT were prospectively enrolled, and outcomes were compared with those of a prospectively registered contemporary surgical cohort using propensity score matching^12^. The trial reported that 3-year overall survival with SBRT was non-inferior to surgery. However, whether SBRT provides long-term outcomes comparable to resection has not been fully determined, largely because robust randomized controlled trial data are lacking. Consequently, surgical resection is generally preferred when feasible, whereas SBRT remains an important option for patients with high operative risk or who refuse surgery^9–11^.

Surgical management of early-stage NSCLC has also advanced. The JCOG0802/WJOG4607L trial demonstrated that segmentectomy improved overall survival compared with lobectomy in patients with small peripheral NSCLC^17^. Similarly, the CALGB/Alliance 140503 trial confirmed that sublobar resection (segmentectomy or wedge resection) provided non-inferior disease-free survival compared with lobectomy for small peripheral NSCLC^18^. Sublobar resection may reduce treatment-related complications in older patients or those with limited physiologic reserve^19,20^. Furthermore, minimally invasive techniques, including video- and robot-assisted thoracic surgery, have further reduced surgical burden^21,22^, potentially broadening surgical candidacy. These surgical advances may influence the clinical role and utilization of SBRT.

Studies using real-world data to describe treatment patterns provide insight into how clinical advances are implemented in routine practice. Such evidence is essential for clinicians and patients making treatment decisions and for local policy and service planning. In Japan, however, nationwide data on radiotherapy utilization for early-stage NSCLC are limited. This study aimed to address this gap by describing patterns and trends in radiotherapy use among older patients with early-stage NSCLC using nationwide data collected from hospital-based cancer registries.

## Methods

### Data and study population

This observational study used nationwide data collected from hospital-based cancer registries in Japan. The database, maintained by the National Cancer Center, primarily collects information from cancer care hospitals designated by the Ministry of Health, Labour and Welfare, along with other participating institutions. In 2017, it covered > 70% of patients newly diagnosed with cancer in Japan^23^. The registry contains patient-level data on year of diagnosis, age at diagnosis, sex, tumor site and histology based on the International Classification of Diseases for Oncology, Third edition (ICD-O-3), tumor-node-metastasis (TNM) classification according to the Union for International Cancer Control (UICC), and initial treatment. Initial treatment data include whether patients received surgery, radiotherapy, or systemic therapy, but do not include treatment intent or technical details such as specific surgical procedures (e.g., sublobar resection) or radiotherapy techniques (e.g., SBRT).

We identified patients with lung cancer (ICD-O-3 site code C34) diagnosed between 2013 and 2022 who received initial treatment, including observation, at the reporting institution. Eligible patients were aged ≥ 65 years, had epithelial tumor histology, and had clinical TisN0M0 or T1–2N0M0 tumors. Histological classification was determined according to the Rare Cancer Classification table from the SEER Program^24^. The 7th edition of the UICC TNM classification was used for 2013–2017, and the 8th edition for 2018–2022. Patients with clinical Tis tumors were included because some tumors classified as clinical T1 in the 7th edition would now be considered Tis under the 8th edition^25^.

Exclusion criteria were poorly differentiated endocrine carcinoma histology and missing data on surgery or radiotherapy. In Japan, SBRT is indicated for lung cancers ≤ 5 cm; therefore, cases with clinical T2b (UICC 7th edition) were excluded when T2 subclassification was available (2016–2017). Given that T2 subclassification was not recorded for the 2013–2015 period, cases from this period included cT2b.

This study was approved by the ethics committee of Osaka International Cancer Institute (approval number 24196) and conducted in accordance with the Declaration of Helsinki. The ethics committee of Osaka International Cancer Institute waived the requirement for patient consent because this was a retrospective observational study using existing data without any personally identifiable information, with an opt-out opportunity provided at each participating hospital.

### Outcome

The primary outcome was the proportion of patients receiving each initial treatment type. Initial treatment was classified based on procedures performed at the reporting facility into three categories: surgery, radiotherapy, and other. Because the data lacked treatment intent and technical details, all procedures recorded as surgery and all procedures recorded as radiotherapy were each analyzed as a single category. Surgery and radiotherapy were categorized regardless of whether systemic therapy was received. Patients who received both surgery and radiotherapy were classified under the surgery category. The “other” category included systemic therapy, unspecified procedures, and observation.

### Statistical analyses

Patient and treatment variables included period of diagnosis (earlier: 2013–2017; later: 2018–2022), age group (65–74, 75–84, or ≥ 85 years), sex (female or male), clinical T category (cTis/cT1 or cT2), histological type (adenocarcinoma/adenosquamous carcinoma, squamous cell carcinoma, or other), initial treatment type (surgery, radiotherapy, or other), and facility volume (low, medium, or high). The “other” histology category included cases clinically diagnosed as malignant without pathological confirmation. Facility volume was defined as the median annual number of patients with any cancer type who received initial treatment at each facility during the study period. Volumes were categorized as low (≥ 1 to < 1000 cases), medium (≥ 1000 to < 2000 cases), or high (≥ 2000 cases).

Annual trends in the number and proportion of patients by initial treatment types were summarized by year of diagnosis. Treatment-type proportions were standardized to the 2022 patient population by 5-year age group and sex strata. Binary logistic regression was used to examine the association between specific treatment use and the diagnosis period (later vs. earlier). The dependent variable was the use of a specific treatment (yes/no). For each model, “yes” indicated use of that treatment, and “no” included the remaining categories. The independent variable was the diagnosis period. Odds ratios (ORs) and 95% confidence intervals (CIs) were estimated from both unadjusted models and models adjusted for age group and sex. Subgroup analyses by age group, sex, clinical T category, and facility volume were performed to evaluate the association between radiotherapy use and diagnosis period, with models adjusted for age group and sex, where applicable.

All analyses were performed using the R software (version 4.2.3; R Foundation for Statistical Computing, Vienna, Austria). Statistical significance was defined as a 95% CI that did not include 1.0.

## Results

**Fig. 1:**
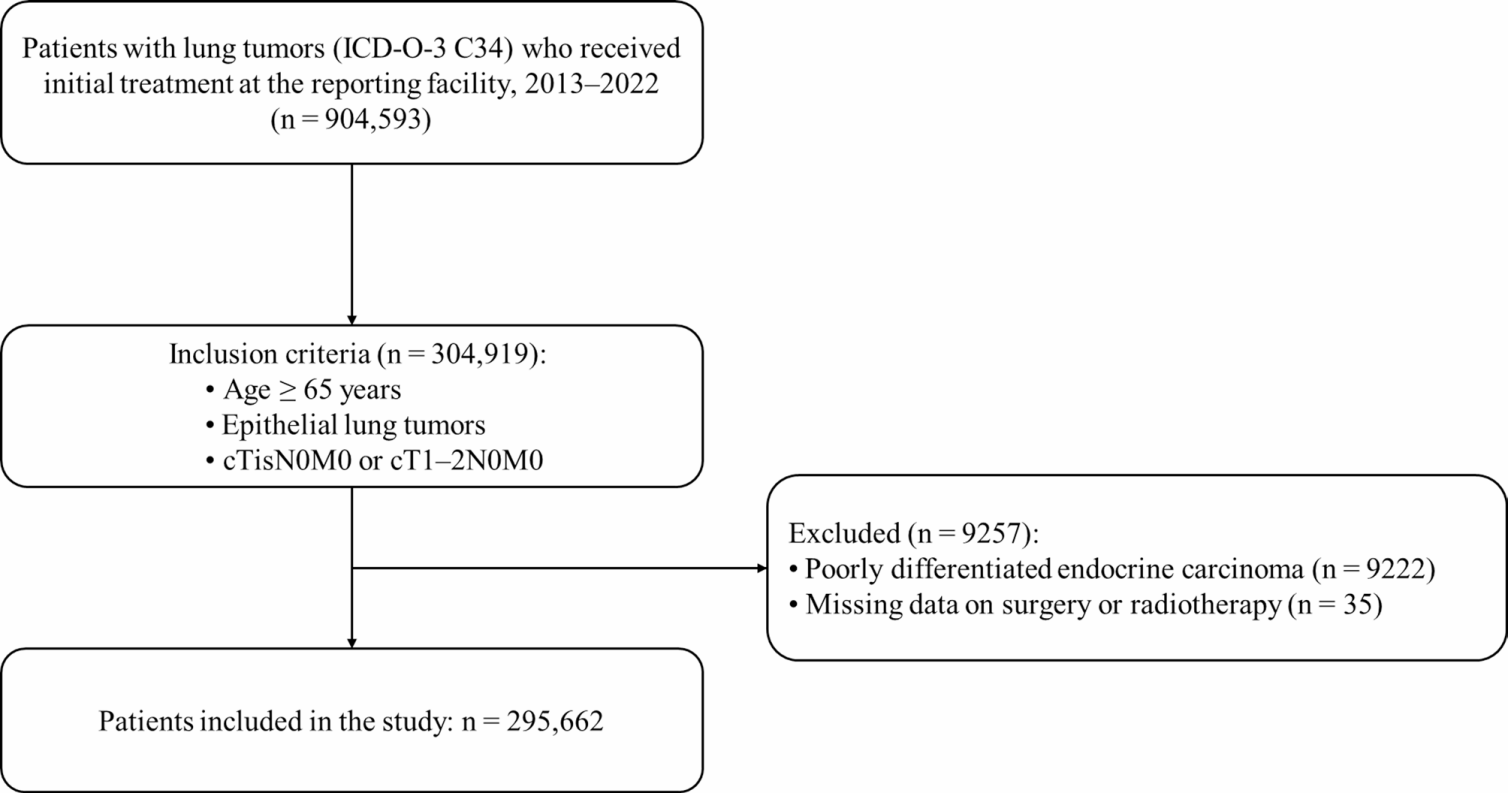
Patient selection flowchart. Initial treatment included observation. Clinical TNM classification followed the UICC TNM system (7th edition, 2013–2017, and 8th edition, 2018–2022). Detailed subclassification of T2 was available from 2016 to 2017, and cases with cT2b during this period were excluded from the study. ICD-O-3, International Classification of Diseases for Oncology, Third edition; UICC, Union for International Cancer Control; TNM, tumor-node-metastasis.

A total of 295,662 patients were included between 2013 and 2022 (Fig. 1, showing patient selection). Of these, 237,517 (80%) underwent surgery, 32,885 (11%) received radiotherapy, and 25,260 (9%) received other types of treatment. The annual number of registered patients increased from 21,805 in 2013 to 35,222 in 2022 (Supplementary Fig. S1, showing annual trends in the number of patients and facilities). The median number of registered patients per hospital increased from 25.0 (interquartile range, 9.2–47.0) in 2013 to 35.0 (interquartile range, 12.0–70.0) in 2022.

The baseline characteristics according to the initial treatment type are summarized in Table 1. Compared with patients who underwent surgery, those who received radiotherapy were more often diagnosed in the later period (59.7% vs. 56.2%), older (median age, 80 vs. 74 years), more often male (69.4% vs. 58.8%), more likely to have cT2 tumors (26.0% vs. 21.8%), and more often treated at high-volume facilities (36.0% vs. 32.9%) but less often at low-volume facilities (21.6% vs. 25.1%). The surgery group had a higher proportion of adenocarcinoma or adenosquamous carcinoma (79.4%), whereas other histological subtypes were more frequent in the radiotherapy group (50.1%).

**Table 1:** Patient characteristics by initial treatment type.

Characteristic	Overall, *N* = 295,662	Surgery, *N* = 237,517	Radiotherapy, *N* = 32,885	Other, *N* = 25,260
**Diagnosis period**				
2013–2017 (earlier)	128,184 (43.4%)	104,011 (43.8%)	13,259 (40.3%)	10,914 (43.2%)
2018–2022 (later)	167,478 (56.6%)	133,506 (56.2%)	19,626 (59.7%)	14,346 (56.8%)
**Median age at diagnosis, years**	75 (70, 79)	74 (70, 78)	80 (75, 84)	81 (75, 85)
**Age at diagnosis, years**				
65–74	146,033 (49.4%)	132,794 (55.9%)	7,614 (23.2%)	5,625 (22.3%)
75–84	126,280 (42.7%)	96,545 (40.6%)	17,807 (54.1%)	11,928 (47.2%)
85+	23,349 (7.9%)	8,178 (3.4%)	7,464 (22.7%)	7,707 (30.5%)
**Sex**				
Female	116,268 (39.3%)	97,807 (41.2%)	10,065 (30.6%)	8,396 (33.2%)
Male	179,394 (60.7%)	139,710 (58.8%)	22,820 (69.4%)	16,864 (66.8%)
**Clinical T category**				
cTis	7,153 (2.4%)	6,848 (2.9%)	86 (0.3%)	219 (0.9%)
cT1	219,621 (74.3%)	178,939 (75.3%)	24,244 (73.7%)	16,438 (65.1%)
cT2	68,888 (23.3%)	51,730 (21.8%)	8,555 (26.0%)	8,603 (34.1%)
**Histology**				
Adeno/adenosquamous	204,040 (69.0%)	188,510 (79.4%)	9,613 (29.2%)	5,917 (23.4%)
Squamous	55,614 (18.8%)	44,982 (18.9%)	6,802 (20.7%)	3,830 (15.2%)
Other	36,008 (12.2%)	4,025 (1.7%)	16,470 (50.1%)	15,513 (61.4%)
**Facility volume**				
Low	77,008 (26.0%)	59,705 (25.1%)	7,097 (21.6%)	10,206 (40.4%)
Medium	123,060 (41.6%)	99,642 (42.0%)	13,945 (42.4%)	9,473 (37.5%)
High	95,594 (32.3%)	78,170 (32.9%)	11,843 (36.0%)	5,581 (22.1%)
**Treatment details**				
Surgery	236,295 (79.9%)	236,295 (99.5%)	—	—
Surgery + radiotherapy	1,222 (0.4%)	1,222 (0.5%)	—	—
Radiotherapy	31,942 (10.8%)	—	31,942 (97.1%)	—
Radiotherapy + systemic therapy	943 (0.3%)	—	943 (2.9%)	—
Systemic therapy	3,035 (1.0%)	—	—	3,035 (12.0%)
Unspecified	643 (0.2%)	—	—	643 (2.5%)
Observation	21,582 (7.3%)	—	—	21,582 (85.4%)

Data are presented as the median (interquartile range) or n (%). The other histology category included cases clinically diagnosed as malignant without pathological confirmation.

The baseline characteristics according to the diagnosis period are summarized in Table 2. Compared with patients diagnosed in the earlier period, those diagnosed in the later period were older with a higher proportion of patients aged ≥ 75 years (52.4% vs. 48.3%). The distribution of clinical T category differed between periods, with higher proportions of cTis (4.0% vs. 0.3%) and cT1 tumors (76.0% vs. 72.1%) and a lower proportion of cT2 tumors (20.0% vs. 27.6%) in the later period. Regarding histology, squamous cell carcinoma was less frequent (17.9% vs. 20.0%), whereas other histological subtypes were more common (13.5% vs. 10.5%) in the later period.

**Table 2:** Patient characteristics by diagnosis period.

Characteristic	Overall, *N* = 295,662	Earlier period (2013–2017), *N* = 128,184	Later period (2018–2022), *N* = 167,478
**Median age at diagnosis, years**	75 (70, 79)	74 (70, 79)	75 (71, 80)
**Age at diagnosis, years**			
65–74	146,033 (49.4%)	66,249 (51.7%)	79,784 (47.6%)
75–84	126,280 (42.7%)	52,782 (41.2%)	73,498 (43.9%)
85+	23,349 (7.9%)	9,153 (7.1%)	14,196 (8.5%)
**Sex**			
Female	116,268 (39.3%)	50,111 (39.1%)	66,157 (39.5%)
Male	179,394 (60.7%)	78,073 (60.9%)	101,321 (60.5%)
**Clinical T category**			
cTis	7,153 (2.4%)	392 (0.3%)	6,761 (4.0%)
cT1	219,621 (74.3%)	92,415 (72.1%)	127,206 (76.0%)
cT2	68,888 (23.3%)	35,377 (27.6%)	33,511 (20.0%)
**Histology**			
Adeno/adenosquamous	204,040 (69.0%)	89,054 (69.5%)	114,986 (68.7%)
Squamous	55,614 (18.8%)	25,687 (20.0%)	29,927 (17.9%)
Other	36,008 (12.2%)	13,443 (10.5%)	22,565 (13.5%)
**Facility volume**			
Low	77,008 (26.0%)	32,572 (25.4%)	44,436 (26.5%)
Medium	123,060 (41.6%)	52,794 (41.2%)	70,266 (42.0%)
High	95,594 (32.3%)	42,818 (33.4%)	52,776 (31.5%)
**Initial treatment**			
Surgery	237,517 (80.3%)	104,011 (81.1%)	133,506 (79.7%)
Radiotherapy	32,885 (11.1%)	13,259 (10.3%)	19,626 (11.7%)
Other	25,260 (8.5%)	10,914 (8.5%)	14,346 (8.6%)

Data are presented as the median (interquartile range) or n (%).

**Fig. 2:**
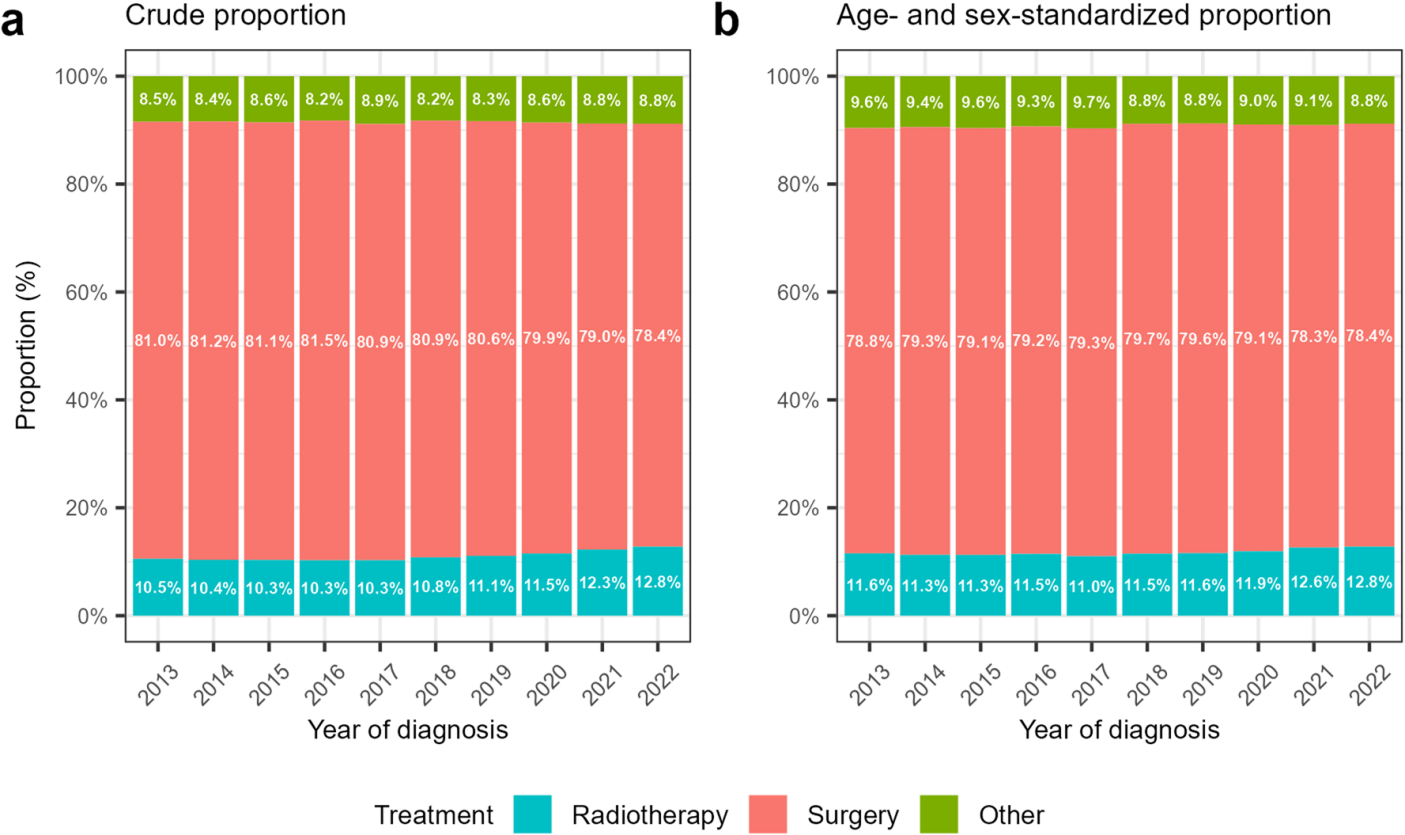
Annual trends in the proportions of initial treatment types: **(a)** Crude proportion; **(b)** Age- and sex-standardized proportion.

The annual trends in the initial treatment types are shown in Fig. 2. Between 2013 and 2022, the crude proportion of patients receiving radiotherapy increased from 10.5% to 12.8% (Fig. 2a). During the same period, the proportion of patients who underwent surgery decreased from 81.0% to 78.4%, and the proportion who received other treatments increased from 8.5% to 8.8%.

After standardization for age and sex, the proportions changed by + 1.2% points for radiotherapy, − 0.4% points for surgery, and − 0.8% points for other treatments (Fig. 2b). In the logistic regression analyses adjusted for age group and sex (Table 3), patients in the later period were more likely to receive radiotherapy than those in the earlier period (adjusted OR [aOR], 1.09; 95% CI, 1.07–1.12). Patients in the later period were less likely to undergo surgery (aOR, 0.98; 95% CI, 0.96–0.99) or other treatments (aOR, 0.94; 95% CI, 0.91–0.96).

**Table 3:** Logistic regression analyses of the association between specific treatment use and the diagnosis period.

Model	Treatment	OR	95% CI
Unadjusted	Radiotherapy	1.15	1.12–1.18
	Surgery	0.91	0.90–0.93
	Other	1.01	0.98–1.03
Adjusted	Radiotherapy	1.09	1.07–1.12
	Surgery	0.98	0.96–0.99
	Other	0.94	0.91–0.96

Analyses included both unadjusted models and models adjusted for age group and sex. The dependent variable was the use of a specific treatment (yes/no), with “yes” indicating the use of that treatment and “no” encompassing the remaining categories. The independent variable was the diagnosis period (later: 2018–2022 versus earlier: 2013–2017). An OR > 1 indicates that patients in the later period are more likely to receive the treatment, whereas an OR < 1 indicates they are less likely to receive it.

OR, odds ratio; CI, confidence interval.

**Fig. 3:**
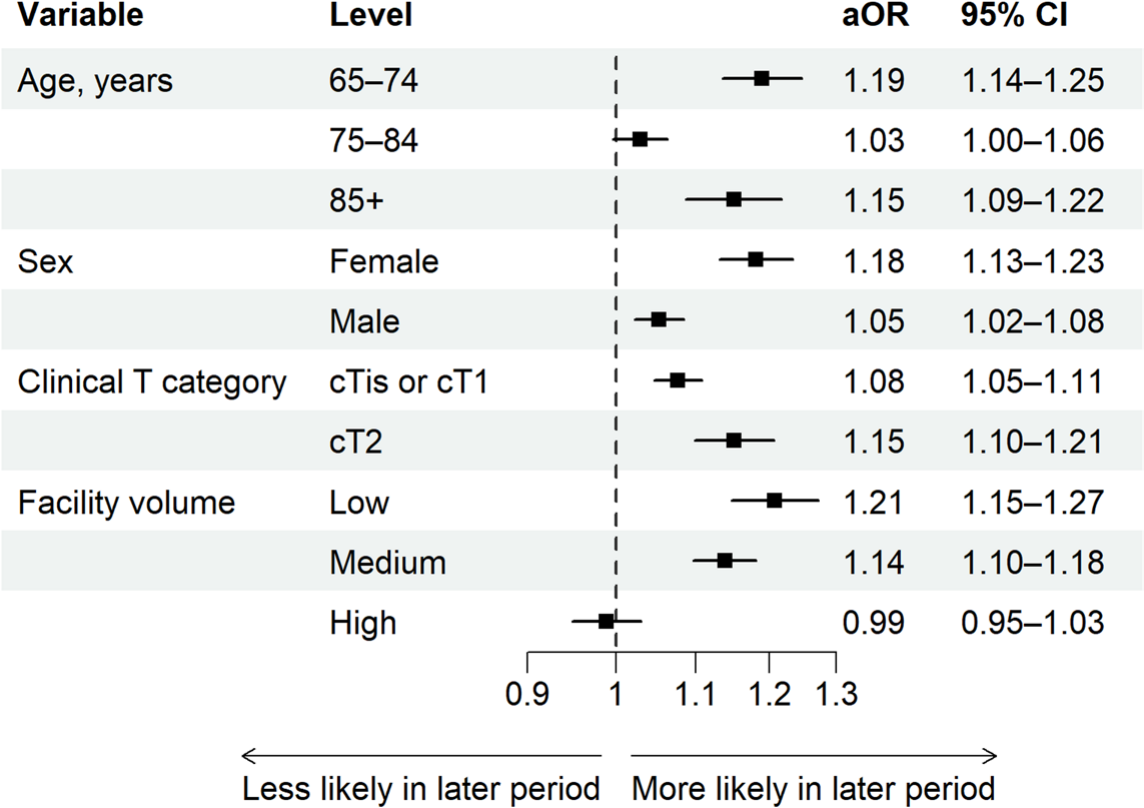
Forest plots of logistic regression subgroup analyses of the association between radiotherapy use and diagnosis period. The models were adjusted for age group and sex when applicable. An aOR > 1 indicates that patients in the later period are more likely to receive radiotherapy, whereas an aOR < 1 indicates that they are less likely to receive it. aOR, adjusted odds ratio; CI, confidence interval.

Figure 3 presents forest plots of the logistic regression subgroup analyses of the association between radiotherapy use and diagnosis period. By age group, patients aged 65–74 years (aOR, 1.19; 95% CI, 1.14–1.25) and those aged ≥ 85 years (aOR, 1.15; 95% CI, 1.09–1.22) had significantly increased odds of radiotherapy use in the later period, whereas no significant change in odds was observed among those aged 75–84 years (aOR, 1.03; 95% CI, 1.00–1.06). By facility volume, significantly increased odds were observed in low-volume (aOR, 1.21; 95% CI, 1.15–1.27) and medium-volume facilities (aOR, 1.14; 95% CI, 1.10–1.18), with no significant change in high-volume facilities (aOR, 0.99; 95% CI, 0.95–1.03). For sex and clinical T category, significantly increased odds were observed across all subgroups.

**Fig. 4:**
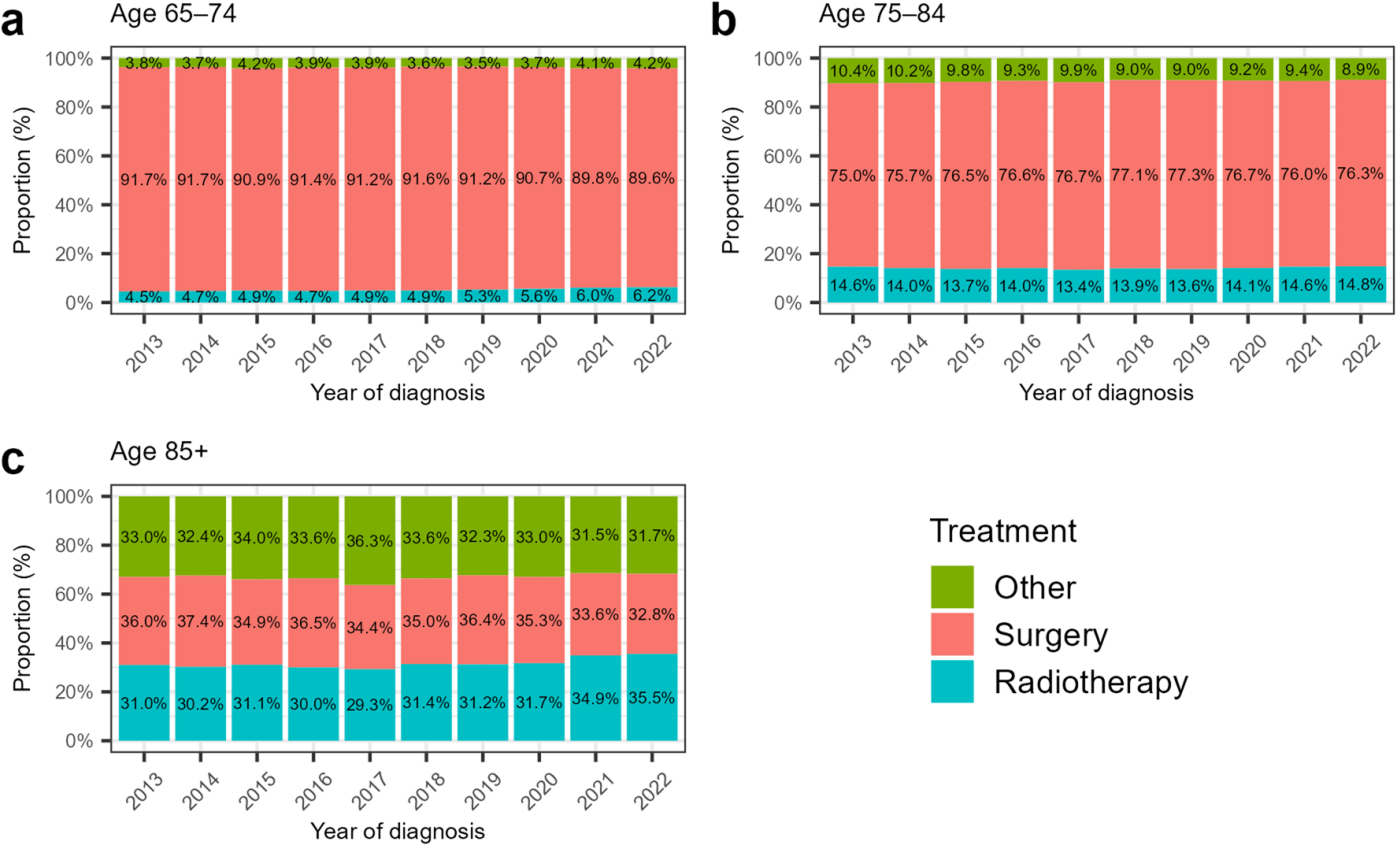
Annual trends in the proportions of initial treatment types stratified by age group: **(a)** 65–74 years; **(b)** 75–84 years; and **(c)** ≥ 85 years.

Figure 4 shows the annual trends stratified by age group (Supplementary Fig. S2 shows the proportions of initial treatment types by 5-year age group for the entire study period). Between 2013 and 2022, the crude proportion of patients receiving radiotherapy changed by + 1.7, + 0.2, and + 4.5% points in the 65–74, 75–84, and ≥ 85 year age groups, respectively, reaching 6.2%, 14.8%, and 35.5% in 2022 (Fig. 4a–c). Over the same period, the proportion of patients who underwent surgery changed by − 2.1, + 1.3, and − 3.2% points in these age groups, with 2022 values of 89.6%, 76.3%, and 32.8%, respectively. The proportion of patients receiving other treatments changed by + 0.4, − 1.5, and − 1.3% points in these age groups, with 2022 values of 4.2%, 8.9%, and 31.7%, respectively. The annual trends by facility volume, sex, and clinical T category are shown in Supplementary Figs. S3–S5.

## Discussion

This study analyzed nationwide trends in radiotherapy use among older patients aged ≥ 65 years with early-stage NSCLC in Japan. Between 2013 and 2022, the crude proportion of patients receiving radiotherapy increased modestly by 2.3% points, reaching 12.8% in 2022. After standardization for age and sex, the increase was attenuated to 1.2% points. The increase in radiotherapy use was not uniform across the age groups. Among patients aged ≥ 85 years, the proportion receiving radiotherapy increased by 4.5% points to 35.5% in 2022, while more than 30% of this age group also underwent surgery during the same year.

Despite accumulating clinical evidence and updated guideline recommendations supporting SBRT^9–13^, the use of radiotherapy for early-stage NSCLC in Japan increased only modestly from 2013 to 2022. This pattern is noteworthy in the context of Japan’s aging population. In contrast, studies from North America have reported a clear increase in SBRT use. In the United States, adjusted analyses showed that SBRT use increased from 1% in 2004 to 20% in 2016, accompanied by declines in both lobectomy and no treatment^14^. A similar pattern was observed in Ontario, Canada, where the proportion of patients receiving SBRT increased from 7.5% in 2010 to 24.4% in 2019^15^. However, a comparison of six European countries and the United States (2010–2015) showed wide international variation in radiotherapy use for early-stage NSCLC, highlighting heterogeneity in practice patterns^26^. Given that previous studies on radiotherapy trends for early-stage NSCLC have been largely limited to Europe and North America, further investigations in other healthcare settings are needed to understand how radiotherapy advances and emerging evidence are reflected in real-world practice.

Several factors may explain this modest increase in Japan. System-level and cultural factors may have limited the expansion of radiotherapy use. Japan’s universal health insurance ensures broad access to medical care, and in our cohort, > 90% of patients at baseline (2013) were already receiving surgery or radiotherapy, leaving little scope for shifts from no treatment to radiotherapy as observed in other countries^14,15^. Cultural preferences may favor surgery over radiotherapy; a survey indicates that patients with cancer often hold negative perceptions of radiotherapy as a curative treatment^27^, and some physicians may share these views. Such attitudes may contribute to a greater use of surgery, even among older patients or those at high surgical risk.

Improvements in physical frailty among older adults in Japan have been reported^28^. Such improvements may have increased the proportion of older patients eligible for surgery, thereby limiting the rise in radiotherapy use. Furthermore, the wider adoption of sublobar resection and minimally invasive techniques may have expanded the surgical candidacy in this population. Annual reports from the Japanese Association for Thoracic Surgery show that, among lung cancer operations, the proportion in patients aged ≥ 80 years increased from 12.5% in 2016 to 16.0% in 2022, and the proportion of sublobar resections increased from 23.7% in 2013 to 36.0% in 2022^29–31^. These trends may explain the slight increase in radiotherapy use among patients aged 75–84 years during the study period.

Notably, > 30% of patients aged ≥ 85 years in our cohort underwent surgery despite increased radiotherapy use, highlighting the persistent uncertainty regarding optimal treatment in the oldest age group (e.g., ≥ 80 or ≥ 85 years). Treatment patterns in this age group vary across countries, but radiotherapy use appears to be increasing^32–35^. Several studies have compared surgery and radiotherapy in this age group.

With respect to oncologic outcomes, although the results vary, surgery appears to yield better long-term outcomes, whereas radiotherapy may provide more favorable short-term survival^34,36–38^. However, the interpretation is complicated by inherent selection bias in nonrandomized studies and potential residual confounding despite statistical adjustment. With respect to treatment-related mortality, differences between surgery and SBRT increase with age, with 30-day mortality reported as 3.94% after surgery versus 0.91% after SBRT in patients aged > 80 years^39^. Regarding quality-adjusted life expectancy, simulation studies suggest that less invasive strategies, including SBRT, may be favorable for some patients aged ≥ 80 years, depending on comorbidity burden and tumor size^40,41^. In older or medically compromised patients, short-term outcomes, such as treatment-related mortality and morbidity, and post-treatment quality of life, may sometimes take precedence over long-term oncologic outcomes^42^. In this context, SBRT may offer advantages, as it is associated with lower treatment-related mortality and morbidity than surgery and typically causes minimal deterioration in quality of life^12,37,39,43–46^.

However, the generalizability of these findings may be limited because treatment outcomes can vary across healthcare settings with different patient characteristics and clinical practices. For example, a prospective observational cohort study from Japan reported a 30-day operative mortality of 1% in patients aged ≥ 80 years with medically operable lung cancer^47^. This lower rate suggests that, in carefully selected patients and within specific healthcare systems, surgical mortality may remain acceptable even at very advanced ages. Accordingly, the relative benefits and risks of surgery versus SBRT in this age group remain unclear, and region-specific comparative studies assessing the comprehensive outcomes are needed.

In the subgroup analyses, significantly increased odds of radiotherapy use in the later period were observed in most of the subgroups. However, no significant association was observed among patients aged 75–84 years, which may reflect the greater use of surgical approaches in this age range, reducing the need for radiotherapy. Additionally, associations were evident at low- and medium-volume facilities but not at high-volume facilities. This difference may reflect the later adoption of SBRT at lower-volume institutions because SBRT requires high-precision techniques and specialized resources. A nationwide survey in Japan reported that 43.3% of responding institutions were equipped for lung SBRT in 2012, whereas 87.8% of responding institutions reported implementing SBRT in 2023^48,49^. As these implementation barriers decreased, more low- and medium-volume facilities may have begun providing SBRT on-site. The present study found a similar proportion of patients receiving radiotherapy across facility volume groups in 2022 (11.3–13.5%; Supplementary Fig. S3).

A strength of this study is its nationwide trend analysis using large-scale data that covers a substantial proportion of patients newly diagnosed with cancer in Japan. The main limitation of this study is the absence of information on treatment intent and radiotherapy techniques. Consequently, the proportions of curative-intent radiotherapy and SBRT are unknown. In addition, clinical information relevant to treatment selection, including pulmonary function, performance status, and comorbidities, is missing. As a result, the actual use of radiotherapy and surgery according to surgical risk cannot be determined. Furthermore, survival comparisons between treatments were not performed because the lack of these clinical variables precludes appropriate adjustment for confounding.

Another limitation is that hospital-based cancer registries primarily include designated cancer care hospitals, introducing potential selection bias at both patient and institutional levels. Patients referred to these hospitals may have greater physiological reserves and may be more likely to receive curative surgery or radiotherapy, potentially leading to overestimation of these treatment proportions compared with the general Japanese population. Standalone radiotherapy centers may also not be consistently captured in these registries, which may bias the estimates toward underestimating radiotherapy use. After the implementation of the Act on the Promotion of Cancer Registries in 2016, the number of participating facilities, mainly low-volume hospitals, increased in 2017, potentially altering the patient characteristics in the registry. To address this, we examined trends across the facility volume groups through subgroup analyses. The 8th edition of the TNM classification for lung cancer introduced measurements based on the size of the solid component^25^. Consequently, among patients classified as cT2 or lower in the later period, a subset was likely to have had a total tumor size > 5 cm. As SBRT in Japan is generally indicated for tumors ≤ 5 cm, patients with a total tumor size > 5 cm may not have been eligible for SBRT.

To address the limitations of national-level coverage and clinical details, studies linking the National Cancer Registry (which covers all cancers in Japan) with radiotherapy-specific databases and insurance claims data are necessary. Such a linkage would enable comparisons of short- and long-term outcomes between surgery and radiotherapy while accounting for comorbidity status, thereby supporting individualized treatment strategies for older patients. The resulting evidence could refine clinical guidelines and inform health policies and service planning for the growing population of older patients with lung cancer.

This nationwide study showed that radiotherapy use for early-stage NSCLC among older patients in Japan increased only modestly over the study period, with surgery remaining the predominant treatment. Given the continued use of surgery even among patients aged ≥ 85 years, comparative studies of surgical and radiotherapy outcomes in this population are needed to clarify the role of radiotherapy in older patients. Given that this study was conducted in Japan, the global generalizability of these findings may be limited. Nevertheless, this study provides new insights into real-world treatment patterns for early-stage NSCLC and supports the importance of region-specific evaluations across healthcare systems and patient populations.

## Supplementary Information

Below is the link to the electronic supplementary material.

Supplementary Material 1

## Data Availability

The data that support the findings of this study are available from the Institute for Cancer Control, National Cancer Center, Japan but restrictions apply to the availability of these data, which were used under license for the current study, and so are not publicly available. Data are however available from the authors upon reasonable request and with permission of the Institute for Cancer Control, National Cancer Center, Japan.
