# Supplementary material for: Nationwide trends in radiotherapy use among older patients with early-stage non-small cell lung cancer in Japan, 2013–2022
Source: Sci Rep. 2026 Mar 31;16:15583. doi: 10.1038/s41598-026-44945-z (PMC13187435; doi:10.1038/s41598-026-44945-z)
Supplement: Supplementary file 1 — Supplementary Material 1 [file 41598_2026_44945_MOESM1_ESM.docx]

**Supporting Information**

**
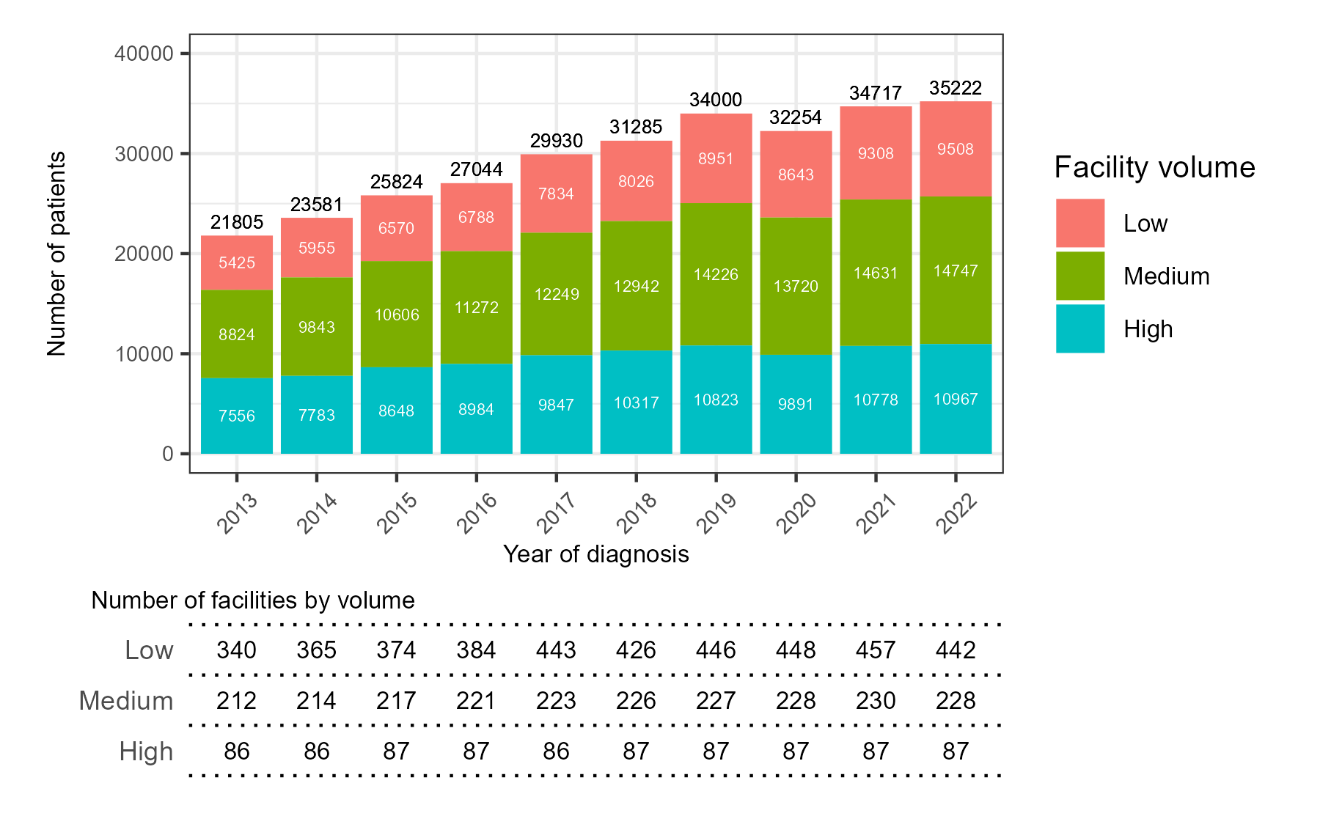
**

**Supplementary Fig. S1.** Annual trends in the number of patients and facilities registering eligible cases. Facility volume was defined as the median annual number of patients with any cancer type who received initial treatment at each facility: low (≥1 to <1000 cases), medium (≥1000 to <2000 cases), or high (≥2000 cases).

**
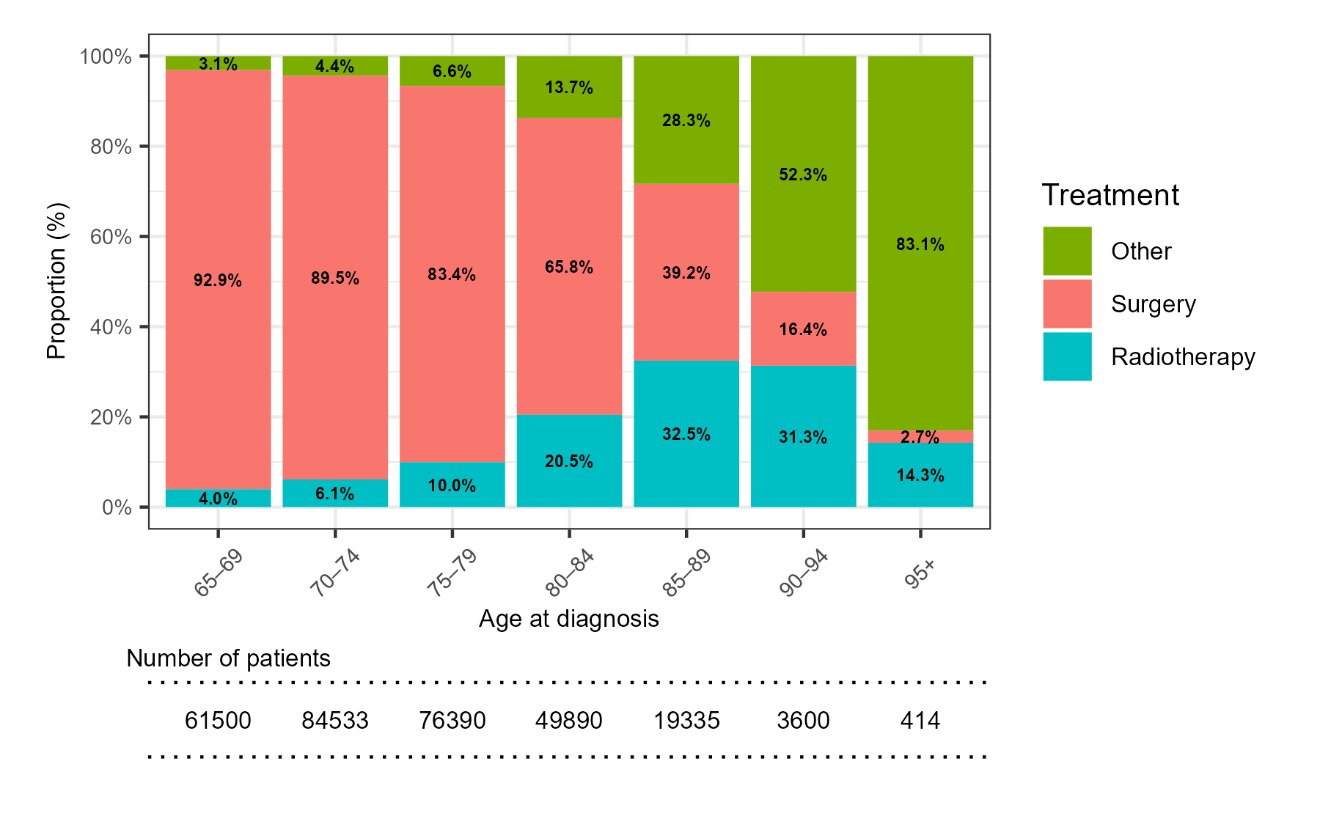
**

**Supplementary Fig. S2.** Proportions of initial treatment types by 5-year age group for the entire study period.

**
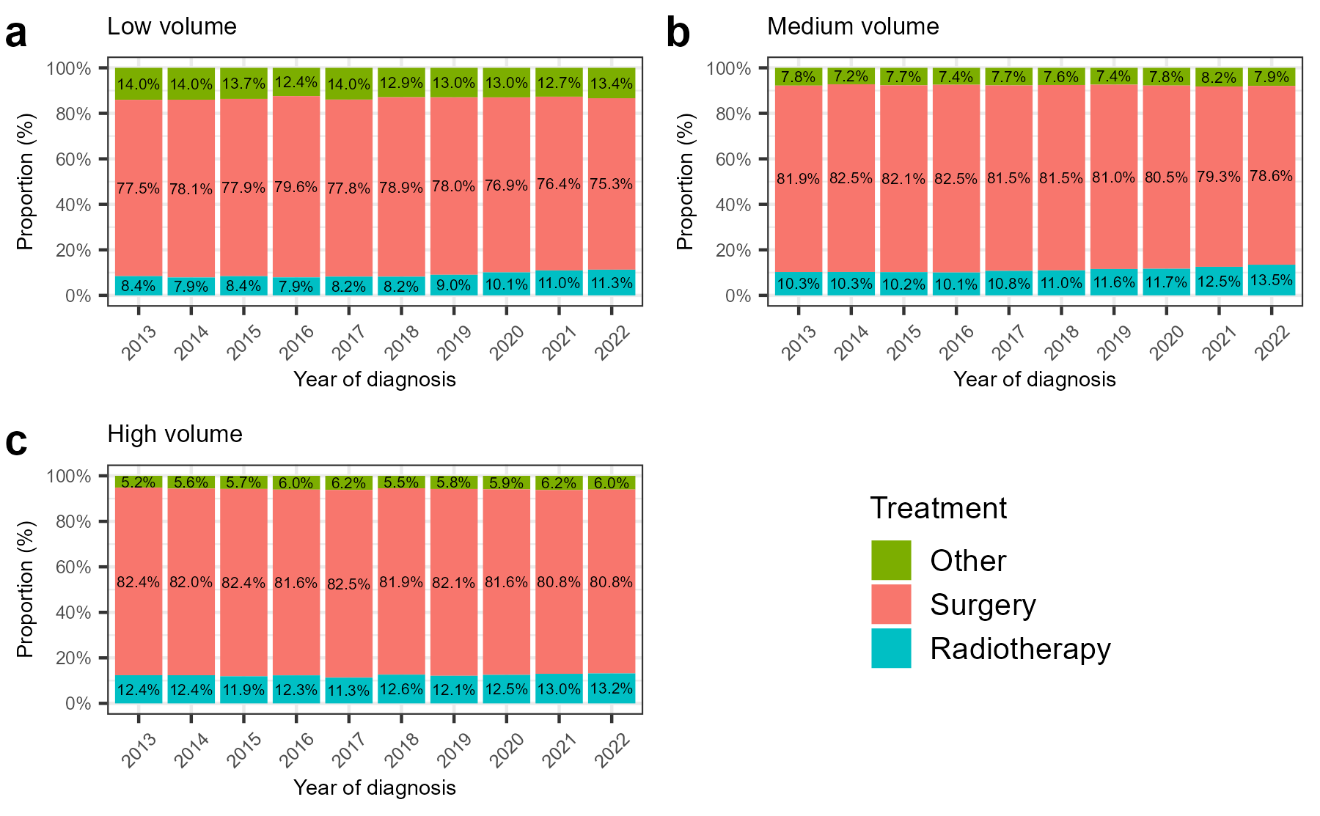
**

**Supplementary Fig. S3.** Annual trends in the proportions of initial treatment types by facility volume: **(a)** Low; **(b)** Medium; and **(c)** High. Facility volume was defined as the median annual number of patients with any cancer type who received initial treatment at each facility during the study period: low (≥1 to <1000 cases), medium (≥1000 to <2000 cases), or high (≥2000 cases).

**
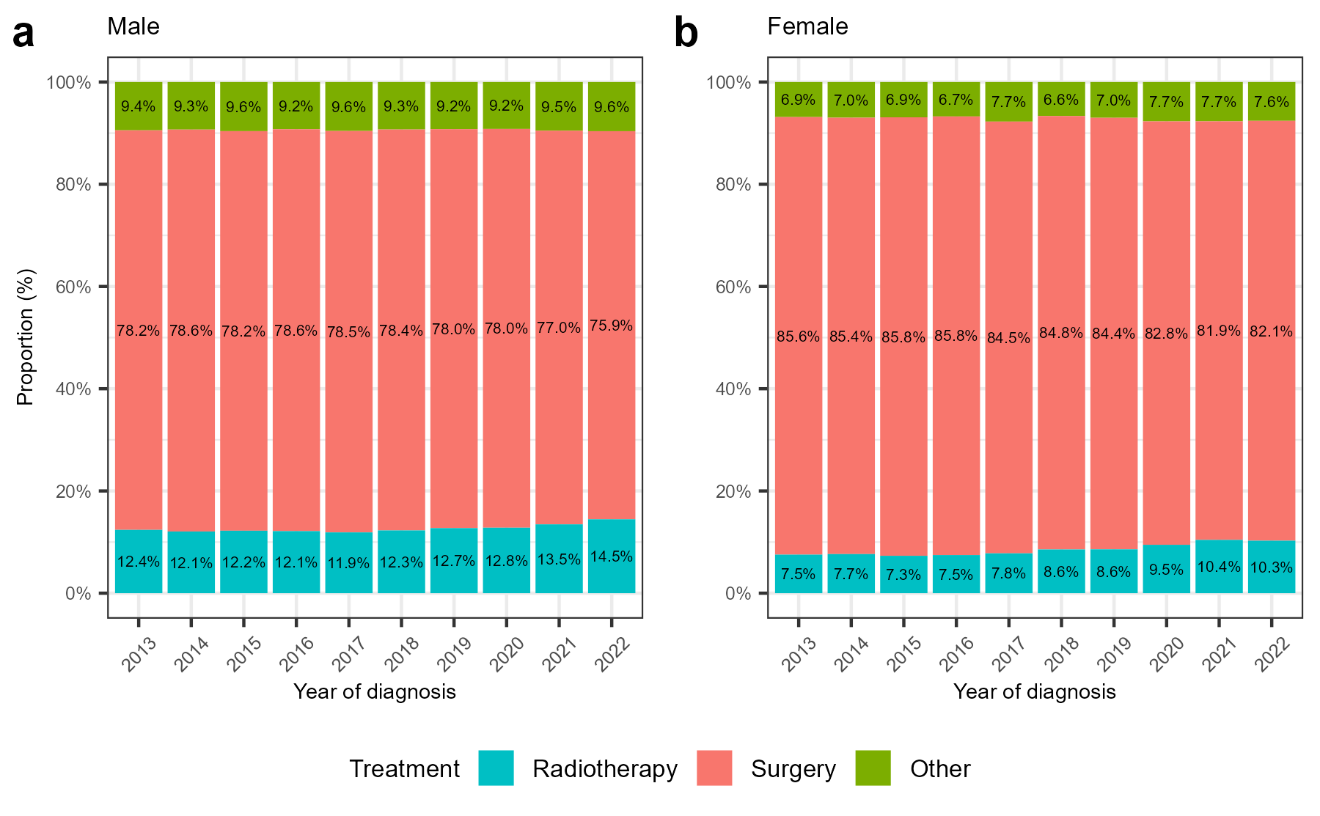
**

**Supplementary Fig. S4.** Annual trends in the proportions of initial treatment types by sex: **(a)** Male; **(b)** Female.

**
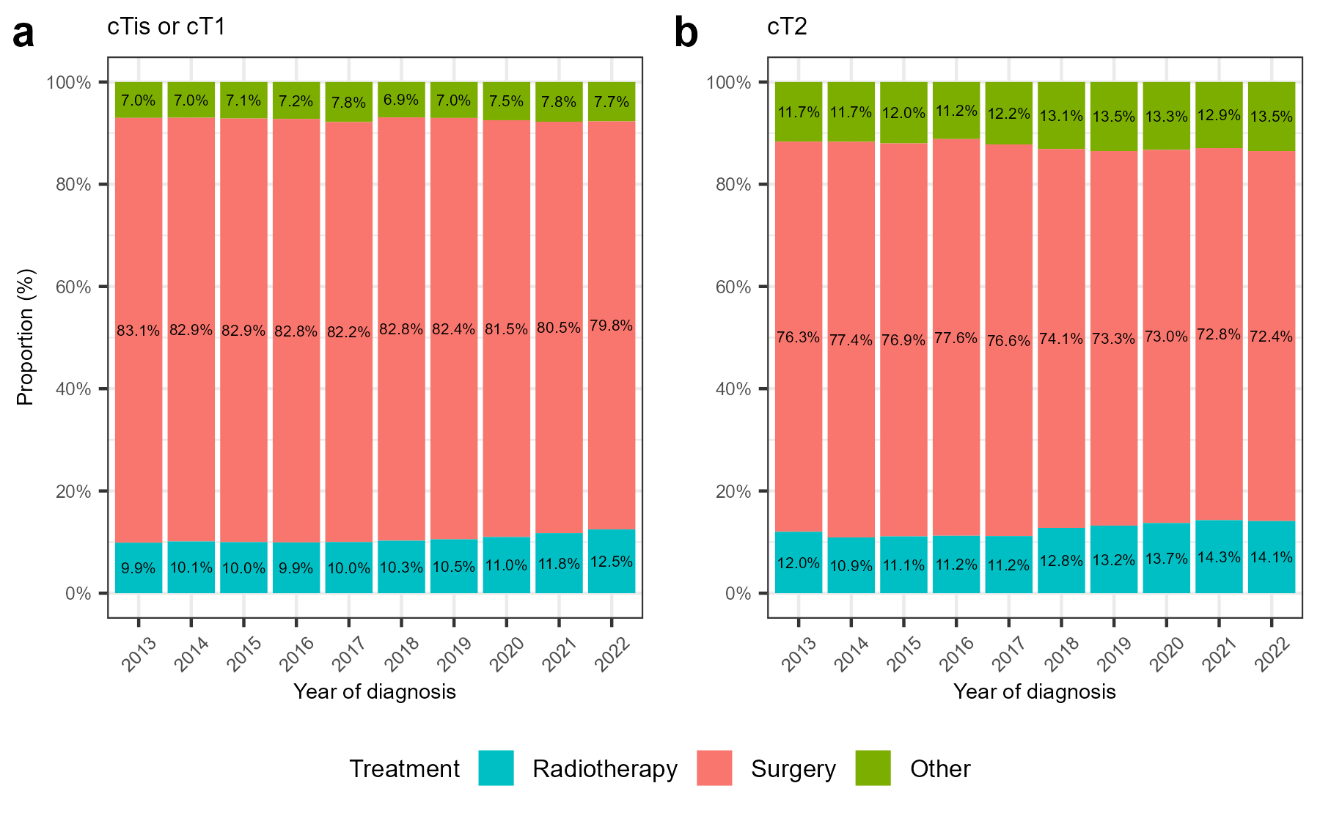
**

**Supplementary Fig. S5.** Annual trends in the proportion of initial treatment types by clinical T category: **(a)** cTis or cT1; **(b)** cT2
